# Bayesian Estimates of Transition Probabilities in Seven Small Lithophytic Orchid Populations: Maximizing Data Availability from Many Small Samples

**DOI:** 10.1371/journal.pone.0102859

**Published:** 2014-07-28

**Authors:** Raymond L. Tremblay, Michael A. McCarthy

**Affiliations:** 1 Department of Biology, University of Puerto Rico – Humacao campus, Humacao, Puerto Rico, United States of America; 2 Catec: Center for Applied Tropical Ecology and Conservation, University of Puerto Rico, Río Piedras, Puerto Rico, United States of America; 3 School of Botany, University of Melbourne, Parkville, VIC, Australia; University of Melbourne, Australia

## Abstract

Predicting population dynamics for rare species is of paramount importance in order to evaluate the likelihood of extinction and planning conservation strategies. However, evaluating and predicting population viability can be hindered from a lack of data. Rare species frequently have small populations, so estimates of vital rates are often very uncertain due to lack of data. We evaluated the vital rates of seven small populations from two watersheds with varying light environment of a common epiphytic orchid using Bayesian methods of parameter estimation. From the Lefkovitch matrices we predicted the deterministic population growth rates, elasticities, stable stage distributions and the credible intervals of the statistics. Populations were surveyed on a monthly basis between 18–34 months. In some of the populations few or no transitions in some of the vital rates were observed throughout the sampling period, however, we were able to predict the most likely vital rates using a Bayesian model that incorporated the transitions rates from the other populations. Asymptotic population growth rate varied among the seven orchid populations. There was little difference in population growth rate among watersheds even though it was expected because of physical differences as a result of differing canopy cover and watershed width. Elasticity analyses of *Lepanthes rupestris* suggest that growth rate is more sensitive to survival followed by growth, shrinking and the reproductive rates. The Bayesian approach helped to estimate transition probabilities that were uncommon or variable in some populations. Moreover, it increased the precision of the parameter estimates as compared to traditional approaches.

## Introduction

A large amount of effort has been placed in understanding population dynamics of orchids [1]–[8], focusing mainly on conservation and management decisions [9]–[13]. Almost all studies are of terrestrial temperate species, whereas approximately 70% of orchid species are epiphytic.

Insufficient information on the basic biology and dynamics of the species often limits attempts to conserve small populations. When population sizes are small, decisions frequently rely on intuition or data from other populations or species. Parameter estimates are usually uncertain when data are limited. In some cases, it is not possible to even construct meaningful credible intervals [14]. Traditional approaches using standard statistical methods for evaluating population parameters can be limiting [14]–[17]. Here we use Bayesian methods to estimate the parameters of an orchid population model by using information from a number of populations simultaneously.

One value of the Bayesian approach is that it takes advantage of *a priori* information and considers the probability distribution for the parameter of interest [18]. Moreover, the 95% Bayesian credible interval for a parameter can be interpreted as intended by most biologists as the interval with a 0.95 probability of containing the true value [19]–[20]. A further advantage of the Bayesian approach is that the parameters of arbitrarily complex models can be estimated with relative ease [17]–[18], [21].

As part of a long-term objective of evaluating alternative approaches for the management of small hyper-dispersed species in lithophytic and epiphytic habitats, we evaluated the transition probabilities of a matrix population model seven riparian populations of a lithophytic tropical orchid from the Caribbean. Specifically we asked 1) what are the transition probabilities among life cycle stages, 2) which of the transitions have the highest and lowest elasticities, 3) what is the expected stable stage distribution, 4) how different are populations from a stable stage distribution, and 5) how different are populations from different riparian environments? In answering these questions, we focused on the size and precision of the parameter estimates.

## Methods

### Model system

The model organism *Lepanthes rupestris* Stimson is an epiphytic and mostly lithophytic (on boulders) endemic to Puerto Rico, and found in the Yunque National Forest (YNF). No specific permits were required for research as this species is neither endangered or protected (no removal or manipulation of plants were performed). For further information contact, El Yunque National Forest, HC-01, Box 13490, Rio Grande, PR, 00745–9625, USA. The study was carried out along two rivers, Quebrada Sonadora (latitude 18° 19′17″ N, longitude 65° 49′ 11″ W) and Quebrada Grande (latitude 18° 19′ 09″ N, longitude 65° 49′ 30″W). These rivers are first order tributaries of the Espíritu Santo River in north-eastern Puerto Rico. The sections studied range between 400 and 500 m in elevation and occur in secondary mature ‘Tabonuco Forest’, dominated by *Dacryodes excelsa* [22].

For the purposes of this study, a population is defined as the set of individuals growing on a single boulder (lithophyte) or tree (epiphyte). For ease of communication we will use the term phorophyte to refer to both (tree and boulder) host types. There is a mean of 45.3±8.1 (s.e.) adults per phorophyte and a mean distance of 4.8 m±1.3 (s.e.) between phorophytes [23]. Most populations have fewer individuals than the mean (Median 26); [6], [23]. Populations are clumped in space, small and separated from each other by short distances. In general, populations on separate phorophytes are genetically distinct and gene flow among populations appears to be limited [24] suggesting that each population has limited interactions with its neighbors. *Lepanthes rupestris* is self-incompatible and is protandrous [25]. Although much effort has been invested to discover the mechanism for transferring the pollinaria, pollinators are unknown for this species. The only known pollinator for one of >800 species of the Neotropical genus is described as a black winged fungus gnat [26] and suggests that pollination occurs through pseudo-copulation by male black winged fungus gnats.

### Description of Field Site

Average annual precipitation is 3460 mm but rainfall is seasonal, with higher values from May through December relative to the January to April period [27]. Water discharge in these streams is highly variable and closely follows precipitation events [28]. Large storms increase water discharge to levels that are much higher than normal and are capable of moving boulders and carrying large pieces of debris downstream [28]. The sections of Quebrada Sonadora are much wider (approx. 10 m) than those of Quebrada Grande (approx. 3 m). Most orchid populations are in the understory in the latter river and receive only indirect sunlight compared to populations along Quebrada Sonadora [29]. The size of plants is directly correlated with light availability in ex situ and in situ experiments and influences directly the production of flowers and consequently male and female reproductive success [29]–[30]. However, plant growth was non-linearly related to light availability with maximum growth occurring in the range 1 to 5 mol m^−2^ day^−1^ [29].

### Data collection

**Figure 1:**
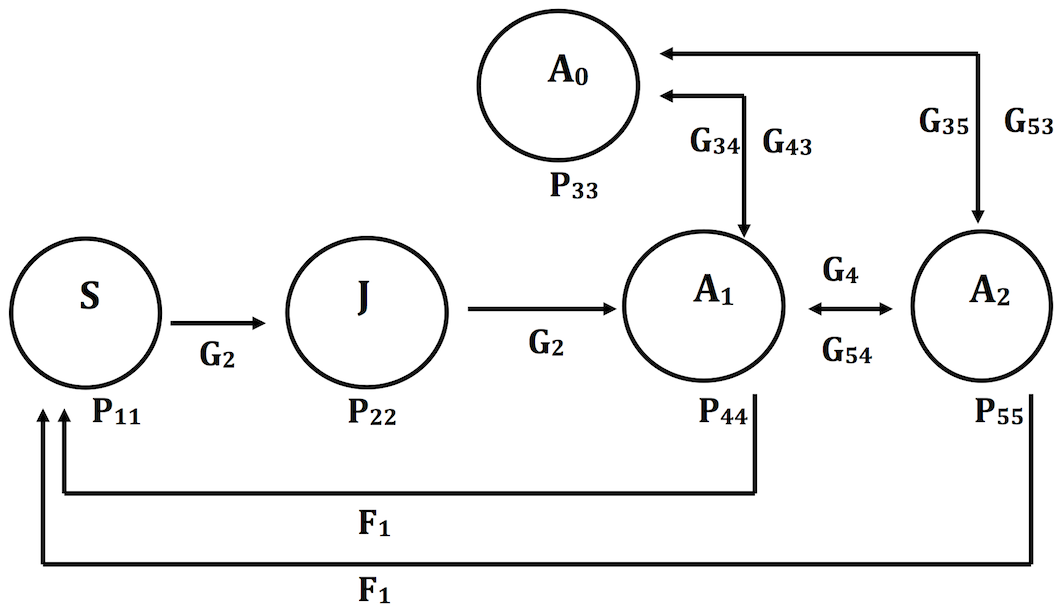
Schematic diagram of the life-cycle of *Lepanthes rupestris* and transitions between life-history stages between successive census dates (monthly surveys). G =  probability of passing from one stage to another, P =  probability of surviving the time period and remaining in the same stage. Arrows labelled with G_ij_'s indicate movement of an individual from one stage to another. P_ij_'s indicate individuals remaining in the same stage at the next census date. The arrow labelled F_ij_ indicates the production of new individuals by sexual reproduction by a specific stage. S =  seedlings, J =  juveniles, A_0_ is a non-reproducing adult stage, representing individuals that have reproduced at some point in their life but which are presently not reproducing, A_1_ =  small reproductive adults (only one active inflorescence), A_2_ =  large reproductive adults (two or more active inflorescence). New individuals are introduced in the populations only from reproducing individuals.

A total of 1346 individuals of *L*. *rupestris* in seven populations were tagged and monitored at monthly intervals for 18 months or more throughout the sampling period in two river systems, Quebrada Sonadora and Quebrada Grande. The number of individuals varied among populations (Table 1). Analyses were performed using demographically structured models in which individuals were grouped into distinct classes. Readers unfamiliar with the approach can read Caswell [31] or Akçakaya *et al.* [32] for a brief explanation of the methods and assumptions. *Lepanthes rupestris* is a small caespitose species of approximately to 15 cm tall; individuals have mainly 2 to 6 slender stems with a solitary coriaceous leaf [33]. Inflorescences are produced and are active throughout the year. Flower production on an inflorescence is sequential from the base, each flower last approximately one and half week (dependent of the environment) and fruits (globose ca. 4 mm; with thousands of seeds) are persistent for 6 weeks. At each survey, individuals were recorded as being in one of five stages, defined as follows (Figure 1): (i) seedlings: small plants without petioles on any leaf, (ii) juveniles: individuals with at least one lepanthiform sheath on the petiole and no current or previously-produced inflorescences (these are persistent) (iii) non-reproducing adults: individuals that were not currently flowering, but carried dried inflorescences from a previous flowering event, (iv) small reproductive adults: individuals with one photosynthesizing (green) inflorescence with or without open flowers or fruits, (v) large reproductive adults: individuals with two or more reproductive shoots. Reproductive effort through female success (fruit set) by all reproducing individuals was noted at every recording date. Fruits remain on plants for about 1.5 months, as each fruit was monitored individually by identifying the leaf, inflorescence and position fruit on the inflorescence production was not overestimated. Consequently, it was possible to account for all fruits produced during the sampled period.

**Table 1:** Number of individuals, and life-history stage at the beginning of the sampling period, in seven populations of *Lepanthes rupestris*.

Population	Months surveyed	Seedlings	Juveniles	Adults
Quebrada Grande 1	34^5^	44	72	97
Quebrada Grande 2	18^4,5^	66	74	95
Quebrada Grande 3	18^2^	107	39	102
Quebrada Sonadora 4	19^3^	40	135	86
Quebrada Sonadora 5	19^3^	14	8	74
Quebrada Sonadora 6	19^2^	28	6	62
Quebrada Sonadora 7	19^2^	66	33	98
Total		365	367	614

Populations were visited once per month between September 1994 and November 1996. ^1^Population Quebrada Grande 1 surveyed since March 93. Months not surveyed ^2^June 94, ^3^July 94, ^4^August 94, ^5^July 95.

In *Lepanthes* spp., survivorship [24], [34]–[36] and reproductive effort are highly correlated with plant stage [34]–[35].

### Statistical Analysis

Data on monthly survival and transitions among the adult age classes were analyzed with multinomial models. For example, non-reproductive adults could remain as non-reproductive adults (n), or transition to small (s) or large reproductive adults (l), or die (d). Mortality of adults in stage *i*, month *j* and population *k* (*m_k_*_,*j*,*i*_) was modelled using logistic regression with categorical variables for stage, month and population. The regression took the form 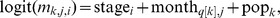
where stage*_i_* is an effect describing how mortality varies among the three adult stages, month*_q_*_[*k*],*j*_ is an effect describing monthly variation in mortality in population *k* that depends on which stream it is in (*q*[*k*] took values of 1 or 2), and pop*_k_* is an effect describing how mortality varies among the different populations. The variable month*_q_*_[*k*],*j*_ was treated as a random effect while the other three variables were treated as fixed effects. Within the two streams, monthly variation in mortality was assumed to be the same for all populations (i.e., within a given month *j*, populations in the same river shared the identical value for the random effect month*_q_*_[*k*],*j*_), while no such correlation was assumed among streams.

Conditional on survival, we assumed that adults reproduced with a particular probability, and that reproductive adults were large or small with a particular probability. These probabilities varied among the type of adult (e.g., adults that were non-reproductive in one month and those that were large reproductive adults had different probabilities of reproducing in the next month) and among populations. However, we assumed that there was not monthly variation in these transitions (conditional on survival). Thus, transitions for an adult of class *i* (taking values n, s, l or d, that specify non-reproductive, small reproductive, large reproductive or dead individuals, respectively) to class n, s, l or d in month *j* and population *k* were specified by the equations
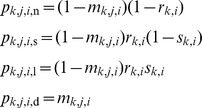
where *r_k_*_,*i*_ is the probability that adults of class *i* in population *k* are reproductive in the next month given that they survive, and *s_k_*_,*i*_ is the probability that adults of class *i* in population *k* are large in the next month given that they survive and become reproductive. We specified, not surprisingly, that dead individuals (*i* = d) remained dead (i.e., *p_k_*_,*j*,*i*,d_ = 0 for *i* = n, s and l, and *p_k_*_,*j*,d,d_ = 1), but estimated the other transition probabilities using the available data.

Data on transitions between adults and the younger stage classes (juveniles and seedlings) and among the younger classes were available on approximately 13-monthly time scales. Conversely, juveniles had sufficient time to develop into small or large reproductive adults, but may remain as juveniles or transition to non-reproductive adults. These assumptions provided realistic limits on the rates of development of individual plants from seedlings to adulthood.

Reproduction rates were estimated using data on occurrence of new seedlings and juveniles in the populations. The data indicated that large reproductive adults produced 7.8 times as many fruits as small reproductive adults and consequently we assumed the ratio was in concordance with the production of seedlings and juveniles. We assumed that this ratio was the same in all populations. Similarly, the ratio of seedlings to juveniles being produced was assumed to be the same in all populations. The actual rate of seedling and juvenile production by reproductive adults was permitted to vary among the seven populations. We assumed that seedlings and juveniles that became non-reproductive adults in a 13-month period (i.e., had evidence of having produced fruits) had contributed to production of seedlings and juveniles at the same rate as small adults. Over this time period, we assumed that seedlings could become small reproductive adults, but did not have sufficient time to become large reproductive adults.

The survival of seedlings and juveniles was modeled using logistic regression, with the rate varying among populations, and between these two stages. Thus, survival of seedlings in population *k* was given by logit(*s*_y,*k*_)  = *a_k_*, and survival of juveniles was given by logit(*s*_j,*k*_)  = *a_k_*+*b*.

Conditional on the seedlings and juveniles surviving, the probabilities of progressing to older stages were expressed as a series of conditional probabilities. Thus, the probability of a seedling (class y) becoming a juvenile (class j) in population *k* was equal to *p_k_*_,y,j_ = *s*_y,*k*_ *g*_y_ *t*_s,j_, where *g*_y_ is the probability of the seedling progressing to an older stage conditional on surviving, and *t*_s,j_ is the probability of a seedling becoming a juvenile conditional on the seedling surviving and not remaining a seedling.

For seedlings, the probability of becoming a non-reproductive adult was *p_k_*_,y,n_ = *s*_y,*k*_ *g*_y_(1–*t*_s,j_)*t*_s,n_, where *t*_s,n_ is the probability of a seedling becoming a non-reproductive adult conditional on it surviving. Finally, the probability of a seedling becoming a small reproductive adult was *p_k_*_,y,n_ = *s*_y,*k*_ *g*_y_(1–*t*_s,j_)(1–*t*_s,n_). Similar expressions were used for the transition probabilities of juveniles to the juvenile, non-reproductive, small reproductive and large reproductive stages.

The parameters of the model were estimated using the observed data on transitions for the seven populations. The estimation procedure was conducted in WinBUGS, a program for conducting Bayesian analyses with Markov chain Monte Carlo methods [37]. This was used because it permitted a relatively easy analysis of the complex hierarchical model [17] and accounted for instances when data were missing. Missing data such as the absence of detection of transition from one stage to another can be estimated for a specific population from the whole data. In addition when events are rare in a population or sample size are small, parameter estimates can be unrealistic if estimated from the data for the single population, hierarchical model uses the whole data set to calculate the best parameter estimates for each specific populations. Consequently, Winbugs allows for complex hierarchical models, which in this case estimates transition probabilities from logistic regression for survival and transitions. Thus one advantage is that it estimates simultaneously the parameters of survival and transitions for all populations while traditional methods estimate these individually for each population [31].

To reflect a lack of prior information, vague prior distributions were used for the parameters. Uniform distributions between zero and one were used for probabilities (e.g., *g*_y_, *t*_y,j_, etc), and normal distributions with means of zero and standard deviations of 1000 were used for the regression coefficients (e.g., *a_k_*, *b*, stage*_i_* and pop*_k_*,). The large standard deviation for the prior meant that it had essentially no influence on the parameter estimate. The random effects for monthly variation in adult mortality (month*_j_*) were modeled as deviates drawn from a normal distribution with a mean of zero and standard deviation that had a wide uniform prior distribution.

Code for conducting the analysis is given in the Appendix. A total of 10000 samples from the posterior distributions of the parameters were taken after discarding the initial 5000 samples as a burn in. The sampled parameter values were exported from WinBUGS and analyzed in the program Mathematica to calculate the asymptotic growth rate (dominant eigenvalue of the 13-monthly transition matrix), stable stage distribution and elasticities of the parameter values as defined by Caswell [31]. These population parameters were calculated within Mathematica for each set of parameter values generated from WinBUGS, and then their mean and standard deviation were calculated to characterize their posterior distributions. Monthly survival rates of adults were converted to 13-monthly rates by raising a sub-matrix of monthly transition probabilities to the power of 13 (i.e., we did not allow multiple reproduction events by adults within a single 13-month period). Our model does not contain density dependence, catastrophes and genetic effects.

For those unfamiliar with the Bayesian approach we recommend that they explore the terminology and methodology with these approachable books [21], [38]–[40].

## Results

### Variation in reproductive potential

Flower production varied greatly among individuals and populations (Table 2). In a few populations most adult individuals produced flowers (pop 5 and 7) while in other populations the number of individuals that failed to produce flowers was significantly higher (pop 1). Pollinaria removal (evidence of pollinator activity) and fruit set showed a similar pattern of large variation among individuals and populations. This suggests that pollinator activity is different among populations.

**Table 2:** Number of fruits, number of recruits, and mean number of recruits produced per reproducing adult, recorded over the whole survey period for seven populations of *Lepanthes rupestris*.

Population	Number of fruits produced	Number of recruits	Mean number of recruits per adult/month
1	198	25	0.0180
2	72	47	0.0399
3	110	41	0.0361
4	105	11	0.0090
5	120	12	0.0111
6	25	5	0.0074
7	126	18	0.0199
All Pop	756	159	0.0206

No relationship was noted between number of fruits produced and recruits in the seven populations surveyed (linear regression, Number of recruits  = 18.462824+0.0393654 *Number of fruits, r^2^ = 2%. F_1,6_ = 0.087, NS).

In all populations larger plants had a higher probability of bearing more fruits than small reproductive adults. A small reproductive plant had a 1 to 4% probability of bearing a fruit in any of the months sampled, while a plant having two or more active inflorescences had a 7 to 20% probability of bearing fruits. In spite of this, most adult plants when reproductive were small, only a very small fraction of reproductive adults had two or more inflorescences (see below, section on stable stage distribution).

### Transition Probabilities

The stage with the highest probability of death during the survey period was dominated by seedlings and was consistent for all populations (10–51%; Table 3 to Table 9). The juveniles and non-reproductive adult stages shared the second highest probability of death, while large reproductive adults were least likely to die (3–20%). The transition probabilities varied among the seven populations. For example, the likelihood of a seedling staying in the same stage in the next time period ranged from 19 to 34%. The stage that appears to impede growth in the life history of *Lepanthes rupestris* is the juvenile stage where, in most of the populations, individuals remained juveniles in the next survey period (48–72%). Large adults did not stay in that stage for long; only in populations one and five was the likelihood of remaining as a large adult greater than 50% while in population three the likelihood of remaining in that stage was much lower (31%). Non-reproductive adults had a high probability of becoming reproductive (flower production) in the next time period in almost all populations except population three, where non-reproductive adults often remained in that stage (Table 3 to Table 9).

**Table 3:** Mean transition probabilities (and standard deviation of the estimate) for a 13-month period for population 1.

	Seedlings	Juveniles	Non-reproducing adults	Small reproductive adults	Large reproductive adults
Seedlings	0.3096 (0.0290)	0.0037 (0.0010)	0	0.0373 (0.0083)	0.0427 (0.0090)
Juveniles	0.4696 (0.0355)	0.6786 (0.0295)	0	0.3025 (0.0645)	0.3333 (0.0701)
Non-reproducing adults	0.0197 (0.0077)	0.0962 (0.0142)	0.0865 (0.0115)	0.0877 (0.0138)	0.0880 (0.0134)
Small reproductive adults	0.0141 (0.0064)	0.0841 (0.0132)	0.1679 (0.0179)	0.1724 (0.0173)	0.1757 (0.0169)
Large reproductive adults	0	0.0194 (0.0064)	0.5931 (0.0404)	0.6166 (0.0362)	0.6389 (0.0326)

**Table 4:** Mean transition probabilities (and standard deviation of the estimate) for a 13-month period for population 2.

	Seedlings	Juveniles	Non-reproducing adults	Small reproductive adults	Large reproductive adults
Seedlings	0.2043 (0.0247)	0.0051 (0.0012)	0	0.07114 (0.0117)	0.0783 (0.012734)
Juveniles	0.3091 (0.0327)	0.5078 (0.0374)	0	0.5543 (0.0910)	0.6109 (0.0992)
Non-reproducing adults	0.0130 (0.0051)	0.0720 (0.0114)	0.2892 (0.0236)	0.2883 (0.0235)	0.2877 (0.0232)
Small reproductive adults	0.0093 (0.0042)	0.0630 (0.0106)	0.3163 (0.0175)	0.3196 (0.0170)	0.3216 (0.0167)
Large reproductive adults	0	0.0145 (0.0049)	0.3452 (0.0236)	0.3518 (0.0235)	0.3558 (0.0235)

**Table 5:** Mean transition probabilities (and standard deviation of the estimate) for a 13-month period for population 3.

	Seedlings	Juveniles	Non-reproducing adults	Small reproductive adults	Large reproductive adults
Seedlings	0.3220 (0.0272)	0.0122 (0.0027)	0	0.1239 (0.0221)	0.1365 (0.0235)
Juveniles	0.4856 (0.0303)	0.6937 (0.0240)	0	0.9964 (0.1724)	1.0644 (0.1836)
Non-reproducing adults	0.0204 (0.0079)	0.0983 (0.0143)	0.3195 (0.0226)	0.3213 (0.0222)	0.3214 (0.0219)
Small reproductive adults	0.0146 (0.0065)	0.0860 (0.0133)	0.3126 (0.0189)	0.3150 (0.1851)	0.3166 (0.0182)
Large reproductive adults	0	0.0198 (0.0065)	0.2976 (0.0254)	0.3026 (0.0256)	0.3104 (0.0262)

**Table 6:** Mean transition probabilities (and standard deviation of the estimate) for a 13-month period for population 4.

	Seedlings	Juveniles	Non-reproducing adults	Small reproductive adults	Large reproductive adults
Seedlings	0.3413 (0.0297)	0.0028 (0.001)	0	0.0278 (0.0083)	0.0307 (0.0092)
Juveniles	0.5181 (0.0338)	0.7227 (0.0255)	0	0.2171 (0.0651)	0.2393 (0.0714)
Non-reproducing adults	0.0217 (0.0084)	0.1024 (0.0149)	0.1542 (0.0177)	0.1554 (0.0174)	0.1550 (0.0170)
Small reproductive adults	0.0156 (0.0070)	0.0896 (0.0139)	0.2979 (0.0218)	0.3017 (0.0211)	0.3043 (0.0206)
Large reproductive adults	0	0.0207 (0.0068)	0.4512 (0.0315)	0.4617 (0.0308)	0.4749 (0.0302)

**Table 7:** Mean transition probabilities (and standard deviation of the estimate) for a 13-month period for population 5.

	Seedlings	Juveniles	Non-reproducing adults	Small reproductive adults	Large reproductive adults
Seedlings	0.3219 (0.0394)	0.0017 (0.0006)	0	0.0174 (0.0051)	0.0191 (0.0055)
Juveniles	0.4887 (0.0520)	0.6949 (0.0478)	0	0.1354 (0.0398)	0.1491 (0.0430)
Non-reproducing adults	0.0205 (0.0081)	0.0985 (0.0154)	0.1474 (0.0218)	0.1489 (0.0210)	0.1495 (0.0204)
Small reproductive adults	0.0147 (0.0067)	0.0861 (0.0143)	0.2085 (0.0197)	0.2144 (0.0187)	0.2185 (0.0182)
Large reproductive adults	0	0.0199 (0.0066)	0.4982 (0.0395)	0.5202 (0.0364)	0.5365 (0.0341)

**Table 8:** Mean transition probabilities (and standard deviation of the estimate) for a 13-month period for population 6.

	Seedlings	Juveniles	Non-reproducing adults	Small reproductive adults	Large reproductive adults
Seedlings	0.2638 (0.0366)	0.0006 (0.0005)	0	0.0070 (0.0050)	0.0077 (0.0055)
Juveniles	0.4008 (0.0502)	0.6085 (0.0555)	0	0.0543 (0.0391)	0.0598 (0.0427)
Non-reproducing adults	0.0168 (0.0068)	0.0862 (0.0146)	0.1841 (0.0274)	0.1925 (0.0263)	0.1986 (0.0266)
Small reproductive adults	0.0120 (0.0056)	0.0754 (0.0133)	0.2255 (0.0281)	0.2366 (0.0263)	0.2450 (0.0248)
Large reproductive adults	0	0.0174 (0.0059)	0.3272 (0.0413)	0.3459 (0.0400)	0.3611 (0.0391)

**Table 9:** Mean transition probabilities (and standard deviation of the estimate) for a 13-month period for population 7.

	Seedlings	Juveniles	Non-reproducing adults	Small reproductive adults	Large reproductive adults
Seedlings	0.1881 (0.0245)	0.0017 (0.0005)	0	0.0249 (0.0061)	0.0274 (0.0066)
Juveniles	0.2856 (0.0335)	0.4783 (0.0462)	0	0.1942 (0.0479)	0.2138 (0.0518)
Non-reproducing adults	0.0120 (0.0048)	0.0678 (0.0116)	0.1215 (0.0173)	0.1268 (0.0168)	0.1313 (0.0164)
Small reproductive adults	0.0086 (0.0040)	0.0593 (0.0107)	0.1887 (0.0218)	0.1972 (0.0205)	0.2047 (0.0193)
Large reproductive adults	0	0.0137 (0.0047)	0.4401 (0.0440)	0.4611 (0.0406)	0.4802 (0.0376)

### Asymptotic population growth rates of individual populations

**Figure 2:**
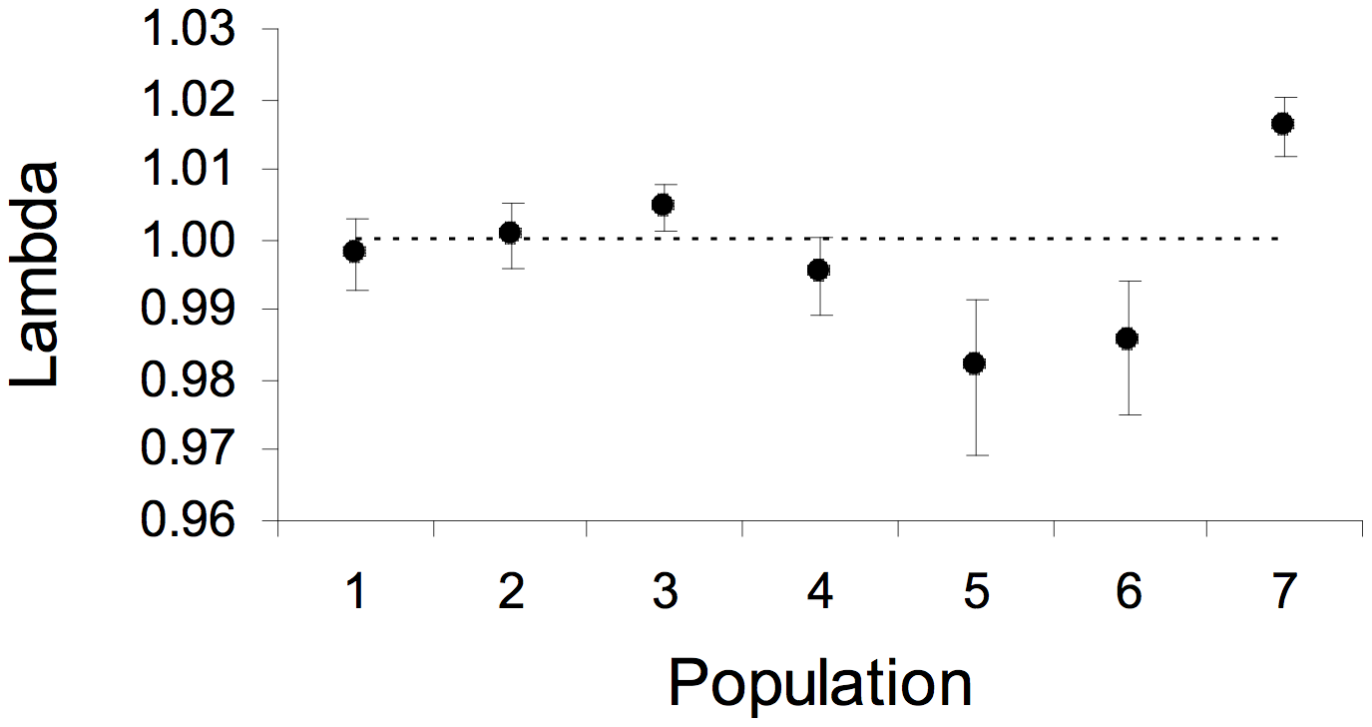
Asymptotic growth rate of the seven populations of *Lepanthes rupestris*. The circles are the mean of the posterior distribution and the bars represent the 95% credible intervals.

The asymptotic population growth rates of *Lepanthes rupestris* had a range of 0.98 to 1.01 (Figure 2). In three of the populations the asymptotic population growth rate cannot be distinguished from stability (1, 4 and 5) because the 95% credible interval overlapped 1.00, while two populations had asymptotic growth rate below one by approximately 1–2% (5 and 6) and two populations had asymptotic growth rates above one (2 and 3). The positive growth in population 2 is small and suggests a maximum increase of less then 1%, while population 3 was increasing by about 1–2%.

Sites on both river systems (Quebrada Grande, pop 1–3; Quebrada Sonadora, pop 4–7) had similar population growth rates (Fig. 2), although the highest population growth rate occurred on Quebrada Grande, the shadier river, and the two lowest population growth rates occurred on Quebrada Sonadora.

### Elasticities

In all populations, modifying the parameters for recruitment (F_ij_), survivorship (P_ij_) and growth (G_ij_) of seedlings would have little impact on population growth rates as most elasticities are small (elasticities ≤0.005; Table 10 to Table 16). In almost all cases the largest elasticities were observed for the proportion of large reproductive adults remaining reproductive (with a maximum elasticity of 0.276). In six of the populations, the largest elasticities are associated with the larger class sizes of adults, and mainly influenced by small and large reproductive adults remaining reproductive and growing to the larger size class. The exception is population three where the proportion of juveniles remaining juveniles has the largest elasticity, with low values for all other transitions. The sum of the elasticities of the small and large adults was comprised between 0.59 to 0.71, except for population three where it comprised a smaller fraction (0.46).

**Table 10:** Mean elasticities (with standard deviation) for population 1.

	Seedlings	Juveniles	Non-reproducing adults	Small reproductive adults	Large reproductive adults
Seedlings	0.0038 (0.0007)	0.0007 (0.0002)		0.002 (0.0004)	0.006 (0.0008)
Juveniles	0.008 (0.0011)	0.183 (0.030)		0.023 (0.004)	0.067 (0.007)
Non-reproducing adults	0.0005 (0.0002)	0.042 (0.006)	0.008 (0.002)	0.011 (0.002)	0.030 (0.005)
Small reproductive adults	0.0005 (0.0002)	0.045 (0.008)	0.018 (0.003)	0.027 (0.005)	0.073 (0.009)
Large reproductive adults		0.011 (0.004)	0.065 (0.006)	0.100 (0.007)	0.276 (0.031)

**Table 11:** Mean elasticities (with standard deviation) for population 2.

	Seedlings	Juveniles	Non-reproducing adults	Small reproductive adults	Large reproductive adults
Seedlings	0.001 (0.0003)	0.0003 (0.0001)		0.002 (0.0004)	0.002 (0.0004)
Juveniles	0.004 (0.0007)	0.076 (0.015)		0.038 (0.006)	0.041 (0.006)
Non-reproducing adults	0.0006 (0.0002)	0.037 (0.006)	0.065 (0.011)	0.069 (0.007)	0.067 (0.006)
Small reproductive adults	0.0005 (0.0002)	0.038 (0.007)	0.082 (0.007)	0.089 (0.009)	0.088 (0.007)
Large reproductive adults		0.009 (0.003)	0.091 (0.006)	0.099 (0.007)	0.100 (0.013)

**Table 12:** Mean elasticities (with standard deviation) for population 3.

	Seedlings	Juveniles	Non-reproducing adults	Small reproductive adults	Large reproductive adults
Seedlings	0.005 (0.0007)	0.002 (0.0004)		0.006 (0.0007)	0.005 (0.0006)
Juveniles	0.011 (0.0010)	0.189 (0.016)		0.070 (0.009)	0.059 (0.007)
Non-reproducing adults	0.0009 (0.0003)	0.054 (0.006)	0.050 (0.007)	0.048 (0.005)	0.037 (0.004)
Small reproductive adults	0.001 (0.0004)	0.068 (0.010)	0.070 (0.007)	0.067 (0.007)	0.052 (0.004)
Large reproductive adults		0.016 (0.005)	0.069 (0.006)	0.067 (0.005)	0.053 (0.008)

**Table 13:** Mean elasticities (with standard deviation) for population 4.

	Seedlings	Juveniles	Non-reproducing adults	Small reproductive adults	Large reproductive adults
Seedlings	0.004 (0.001)	0.0006 (0.0002)		0.003 (0.0006)	0.005 (0.0007)
Juveniles	0.008 (0.001)	0.183 (0.029)		0.030 (0.006)	0.045 (0.007)
Non-reproducing adults	0.0004 (0.0002)	0.036 (0.006)	0.019 (0.004)	0.031 (0.005)	0.042 (0.006)
Small reproductive adults	0.0004 (0.0002)	0.037 (0.008)	0.043 (0.005)	0.072 (0.010)	0.097 (0.010)
Large reproductive adults		0.009 (0.003)	0.067 (0.005)	0.112 (0.008)	0.156 (0.022)

**Table 14:** Mean elasticities (with standard deviation) for population 5.

	Seedlings	Juveniles	Non-reproducing adults	Small reproductive adults	Large reproductive adults
Seedlings	0.003 (0.001)	0.0003 (0.0002)		0.002 (0.0005)	0.004 (0.001)
Juveniles	0.006 (0.002)	0.163 (0.054)		0.018 (0.005)	0.042 (0.008)
Non-reproducing adults	0.0003 (0.0001)	0.029 (0.006)	0.020 (0.005)	0.026 (0.005)	0.055 (0.011)
Small reproductive adults	0.0002 (0.0001)	0.029 (0.007)	0.032 (0.005)	0.043 (0.007)	0.093 (0.013)
Large reproductive adults		0.007 (0.003)	0.078 (0.008)	0.108 (0.011)	0.235 (0.036)

**Table 15:** Mean elasticities (with standard deviation) for population 6.

	Seedlings	Juveniles	Non-reproducing adults	Small reproductive adults	Large reproductive adults
Seedlings	0.002 (0.001)	0.0001 (0.0002)		0.001 (0.0008)	0.002 (0.001)
Juveniles	0.003 (0.002)	0.133 (0.111)		0.013 (0.008)	0.020 (0.010)
Non-reproducing adults	0.0001 (0.0001)	0.016 (0.008)	0.044 (0.013)	0.055 (0.014)	0.078 (0.019)
Small reproductive adults	0.0001 (0.0001)	0.016 (0.009)	0.060 (0.013)	0.075 (0.016)	0.107 (0.021)
Large reproductive adults		0.004 (0.003)	0.090 (0.015)	0.114 (0.019)	0.166 (0.040)

**Table 16:** Mean elasticities (with standard deviation) for population 7.

	Seedlings	Juveniles	Non-reproducing adults	Small reproductive adults	Large reproductive adults
Seedlings	0.001 (0.0005)	0.0002 (0.0001)		0.001 (0.0004)	0.003 (0.0008)
Juveniles	0.003 (0.001)	0.082 (0.037)		0.017 (0.005)	0.040 (0.009)
Non-reproducing adults	0.0003 (0.0001)	0.027 (0.007)	0.020 (0.004)	0.029 (0.005)	0.062 (0.010)
Small reproductive adults	0.0003 (0.0001)	0.027 (0.008)	0.035 (0.005)	0.052 (0.008)	0.111 (0.012)
Large reproductive adults		0.007 (0.003)	0.084 (0.007)	0.125 (0.009)	0.273 (0.034)

### The expected stable stage distribution

If populations of *L. rupestris* do attain stability then the expected stable stage distribution of the different populations suggests that the juveniles (in four populations) or the large adults (in three populations) are likely to be the most common of the five stages (Table 17). In all populations, the frequency of seedlings is expected to be small (<1–4%). The expected proportion of reproductive adults (small and large adults) varies extensively among populations from 27–61%. In all populations of Quebrada Grande the juvenile stage comprised the largest proportion of individuals at the stable stage distribution while at Quebrada Sonadora the dominant stage varied among population.

**Table 17:** The expected stable stage distribution of seven populations of lithophytic orchids in the Caribbean national forest.

Population	Mean Seedlings (sd)	Mean Juvenile (sd)	Mean non-reproductive adult (sd)	Mean Small Adult (sd)	Mean Large Adult (sd)
1	0.028 (0.0032)	0.430 (0.039)	0.085 (0.009)	0.125 (0.012)	0.332 (0.034)
2	0.021 (0.004)	0.364 (0.050)	0.127 (0.013)	0.208 (0.021)	0.280 (0.033)
3	0.045 (0.004)	0.542 (0.028)	0.154 (0.015)	0.147 (0.012)	0.112 (0.012)
4	0.021 (0.004)	0.364 (0.050)	0.127 (0.013)	0.208 (0.021)	0.280 (0.033)
5	0.016 (0.003)	0.301 (0.055)	0.134 (0.017)	0.177 (0.017)	0.372 (0.043)
6	0.008 (0.004)	0.175 (0.093)	0.212 (0.029)	0.254 (0.032)	0.352 (0.057)
7	0.022 (0.003)	0.309 (0.053)	0.124 (0.013)	0.177 (0.016)	0.368 (0.040)

The observed frequency distribution at the beginning of the survey was significantly different from the predicted stable stage distribution for all populations (all chi square *p*<0.0001), with the number of observed seedlings higher than expected. The rarity of populations observed at stable stage distribution in epiphytic systems and orchids in general suggest that there is little evidence that these orchids ever attain and sustain stable stage distribution [1]–[7], [24], [34], [36], [41]. It is possible that for many orchid species populations may always be in a transient phase [42]–[44].

## Discussion

### Transition rates

In general a large proportion of seedlings stayed seedlings (19–34%) for more than a year and most juveniles did not growth to the adult stage and remained in the same stage (48–72%). Because of the differences among the width of the two rivers, Quebrada Sonadora and Quebrada Grande, *a priori* we expected life history differences between the two sites. We found that male and female fitness did vary among populations but with no obvious environmental differences [45] except that populations at Quebrada Grande were generally shadier, and likely more humid, than at Quebrada Sonadora, a wider river with more direct sunlight. If pollinators are fungus gnats as we suspect, then local environmental conditions such as humidity, quantity and quality of forest litter, and soil organic matter may control their populations and abundance. Consequently we should see more pollinator activity at Quebrada Grande because of the shadier environment. There was little or no difference in pollinaria removal between the two river systems, but plants at Quebrada Grande experienced higher fruit set. Why did we observe this difference? The answer may rest more with flower response to humidity than any effects on pollinators. Flowers of *Lepanthes* are sensitive to dry conditions; low humidity reduces flower longevity (Tremblay unpublished data). In addition, stigmatic maturity depends on flower age (*L. rupestris* is protandrous), as does the probability of successful pollination and fruit set [25]. Consequently, flowers of Quebrada Sonadora populations may be shorter-lived with reduced opportunity for pollinations. This may explain the tendency for higher asymptotic population growth rates at Quebrada Grande. A constant observed in all populations surveyed is that the transition from juvenile to adulthood is appears to be one of the bottlenecks in the life history of the orchid as this transition was rarely observed during the survey period. Out of a total of 4343 juveniles surveyed (sum of all populations and all months) only 88 of these became adults. What is the cause of this low transition rate to the adult stage but it is likely to be a consequence of multiple environmental (abiotic and biotic interactions) and stochastic factors. The transition between the three adult stages was frequent, and large reproductive individuals were not likely to be in that stage during the next survey period (36–69%). Reproductive effort in all of the *L. rupestris* populations was low and most often limited to a small fraction of adults. Reproductive success is pollinator limited and is highly skewed towards a few individuals [24]. Once the adult stage is attained, mortality is low.

In an extensive review of the population ecology of orchids mediated by environmental factors, Light *et al.* [7] list many studies. However, most of these are short-term studies and descriptive, which fails to use the information to predict population persistence. Most orchid population dynamic studies have been restricted to terrestrial species (for a review see) [46]. Comparative data on epiphytic and lithophytic species are limited to four species [34], [47]–[52].

Large individuals have high reproductive potential and lifespan, but these individuals are rare in the population. The lifetime reproductive success of *Lepanthes* is extremely skewed where most individuals have low (or zero) reproductive success and a few have very high lifetime reproductive success [24]. In an 11-year study of the related *Lepanthes caritensis* Tremblay and Ackerman, some adults may survive for more than 20 years but most individuals live less than 4 years [36]. In many species of orchids, larger individuals have higher reproductive potential or success [53]. There is a correlation between life stages and size of individuals in *Lepanthes*; generally leaf size and leaf number increase as an individual progresses from juveniles to small reproductive adults and large adults. Thus a complicating life history variable is that a large proportion of reproductive orchids become smaller in the next survey period suggesting that flower production or fruit production may be costly. A cost of reproduction has been observed in many species of orchids [53]. In epiphytic species, subsequent plant size (pseudobulb or leaf number or size) is often smaller after a reproduction. In terrestrial species, plants are often dormant the next growth period or emerge as non-reproductive individuals [13],[54], but this is not always the case [55].

In general, survival and growth were the most important demographic processes in all of the populations. Stasis (sum of P_ij_) had the largest of the elasticities in all seven populations (0.10–0.28). While relative contribution of fecundity to change in population growth was negligible for all populations (0.004–0.011). Elasticites of all populations are dominated by growth and survival with fecundity having little impact. This pattern of dominance of elasticities from growth and survival suggest that *Lepanthes rupestris* are more similar to iteroparous forest herbs and not from open habitats [56].

Comparative data from orchids are rare [13],[34],[52],[57]. However, most studies suggest that survivorship is important. For example, in the terrestrial *Ophrys sphegodes* over 87% of the elasticities is explained by survivorship of reproductive individuals [57]. In the epiphytic *Lepanthes caritensis* the highest elasticities are found in stasis of the non-reproductive adults (>0.52%; Tremblay 1997), while in the rare species *Lepanthes eltoroensis* the highest elasticities varied among the five populations. In three populations the survivorship and stasis of non-reproductive adults was the highest and in two populations the stasis of reproductive adults [34]. It appears that promoting flowering production and remaining a reproductive adult would have the largest impact on population growth, however, the relative importance of these cannot be determined *a priori*. In the terrestrial *Prasophyllum correctum* the most important demographic process is the dormant stage (>0.31) [13].

### Bayesian advantage

Our paper illustrates one of the benefits of using of a Bayesian approach for estimating transition probabilities; relatively complex statistical models can be analyzed [17]. These analyses can use information from a wide range of sources simultaneously to fit complex hierarchical models. Because information from several populations is used concurrently, the resulting parameter estimates tend to be more realistic compared to the case where each population is analyzed individually using, for example, traditional methods as in Ebert [58] or Caswell [31]. An example of this is that estimating individual transitions for juveniles for two of the populations was hampered because we observed no deaths during our survey period (data not shown). This result is not unusual when sample sizes are small. Similarly in one of the populations we observed only 1 of 37 (3.7%) individuals that grew from a juvenile to an adult stage, while in another population 38 of 778 (1.3%), which are the best maximum likelihood of the observed data. The Bayesian estimation of this transition can be further refined by taking advantage of all the data from all populations from gave estimates that range from 5.93% to 8.96%, and more importantly, credible intervals of the transition probabilities account for estimation errors. The differences in transition rates could easily be assigned to difference in sample size and not solely as a result of environmental or genetic differences among populations.

## References

[1] Øien DI, Moen A (2002) Flowering and survival of *Dactylorhiza lapponica* and *Gymnadenia conopsea* in Sølendet Nature Reserve, Central Norway. In: Kindlmann P, Willems JH, Whigham DH, editors. Trends and fluctuations and underlying mechanisms in terrestrial orchid populations. Leiden: Backhuys Publishers. pp. 3–22.

[2] Willems JH (2002) A founder population of *Orchis simia* in The Netherlands: a 30-year struggle for survival. In: Kindlmann P, Willems JH, Whigham DH, editors. Trends and fluctuations and underlying mechanisms in terrestrial orchid populations. Leiden: Backhuys Publishers. pp. 23–32.

[3] Tali K (2002) Dynamics of *Orchis ustulata* populations in Estonia. In: Kindlmann P, Willems JH, Whigham DH, editors.Trends and fluctuations and underlying mechanisms in terrestrial orchid populations. Leiden: Backhuys Publishers. pp. 33–42.

[4] Kaitala V, Kull T (2002) Temporal auto-correlation structures in populations of *Cypripedium calceolus*: a two-year rhythm in flowering. In: Kindlmann P, Willems JH, Whigham DH, editors. Trends and fluctuations and underlying mechanisms in terrestrial orchid populations. Leiden: Backhuys Publishers. pp. 43–52.

[5] Shefferson RP (2002) Dormancy and survival in rare terrestrial orchids. In: Kindlmann, P, Willems JH, Whigham DH, editors. Trends and fluctuations and underlying mechanisms in terrestrial orchid populations. Leiden: Backhuys Publishers. pp. 53–64.

[6] Tremblay RL, Meléndez-Ackerman E, Kapan D (2006) Do orchids behave as metapopulations? Evidence from colonization, extinction rates and asynchronous population dynamics. Biol. Cons 129: 70–81.

[7] Light MHS, MacConaill M (2006) Appearance and disappearance of a weedy orchid, *Epipactis helleborine* . Folia Geobot 41: 77–93.

[8] Shefferson RP (2006) Demographic response to shading and defoliation in two woodland orchids. Folia Geobot 41: 95–106.

[9] Carey PD, Farrell L, Stewart NF (2002) The sudden increase in the abundance of *Himantoglossum hircinum* in England in the past decade and what caused it. In: Kindlmann P, Willems JH, Whigham DH, editors. Trends and fluctuations and underlying mechanisms in terrestrial orchid populations. Leiden: Backhuys Publishers. pp. 187–208.

[10] Jersáková J, Kindlmann P, Strítesky M (2002). Population dynamics of *Orchis morio* in the Czech Republic under human influence. In: Kindlmann P, Willems JH, Whigham DH, editors. Trends and fluctuations and underlying mechanisms in terrestrial orchid populations. Leiden: Backhuys Publishers. pp. 209–224.

[11] Dorland E, Willems JH (2002) Light climate and plant performance of *Ophrys insectifera*; a four-year experiment in The Netherlands. In: Kindlmann P, Willems JH, Whigham DH, editors. Trends and fluctuations and underlying mechanisms in terrestrial orchid populations. Leiden: Backhuys Publishers. pp. 225–238.

[12] Primack RB (2002) An eleven year experimental study of the pink lady's slipper orchid (*Cypripedium acaule*). In: Kindlmann P, Willems JH, Whigham DH, editors. Trends and fluctuations and underlying mechanisms in terrestrial orchid populations. Leiden: Backhuys Publishers. pp. 239–249.

[13] Coates F, Lunt I, Tremblay RL (2006) The effect of fire, rainfall, herbivory on population dynamics of a threatened terrestrial orchid from south-eastern Australia, *Prasophyllum correctum* D.L. and consequences for management. Biol. Conser 129: 59–69.

[14] Halpern BS, Regan H, Possingham HP, McCarthy MA (2006) Accounting for uncertainty in marine reserve design. Ecol. Lett 9: 2–11. 16958861 10.1111/j.1461-0248.2005.00827.x

[15] Wade PR (2000) Bayesian methods in conservation biology. Cons. Biol 14: 1308–1316.

[16] Wade PR (2002) Bayesian Population Viability Analysis. In: Beissinger SR, McCullough DR, editors. Population Viability Analysis.Chicago: The University of Chicago Press. pp. 213–238.

[17] Clark JS (2005) Why environmental scientists are becoming Bayesians? Ecol. Lett 8: 2–15.

[18] McCarthy MA, Masters P (2005) Profiting from prior information in Bayesian analyses of ecological data. J. Appl. Ecol 42: 1012–1019.

[19] Berger JO, Berry DA (1988) Statistical analysis and the illusion of objectivity. Am. Sci 76: 159–165.

[20] Bernardo JM, Smith AFM (1994) Bayesian theory. New York: John Wiley and Sons.pp. 610.

[21] McCarthy MA (2007) Bayesian Methods for Ecology. Cambridge: Cambridge University Press. pp. 306.

[22] Waide RB, Reagan DP (1996) The rainforest setting. In: Reagan DP, Waide RB, editors.The food web of a tropical rainforest. Chicago: The University of Chicago Press. pp. 1–16.

[23] Tremblay RL (1997) Distribution and dispersion patterns of individuals in nine species of *Lepanthes* (Orchidaceae). Biotropica 29: 38–45.

[24] Tremblay RL, Ackerman JD (2001) Gene flow and effective population size in *Lepanthes* (Orchidaceae): a case for genetic drift. Biol. J. Linn Soc 72: 47–62.

[25] Tremblay RL, Pomales-Hernández G, Méndez-Cintrón ML (2006) Flower phenology and sexual maturation: Partial protandrous behaviour in three species of orchids. Carib. J. Sci 42: 75–80.

[26] Blanco M, Barboza G (2005) Pseudocopulatory pollination in *Lepanthes* (Orchidaceae: Pleurothallidinae) by fungus gnats. Ann. Bot 95: 763–772. 15728665 10.1093/aob/mci090 PMC4246739

[27] Brown S, Lugo AE, Silander S, Liegel LH (1983) Research opportunities at the Luquillo Experimental Forest. USDA Forest Service Southern Experiment Station General Technical Report SO-44, pp. 128. New Orleans, Louisiana, pp. 128.

[28] Johnson SL, Covich AP, Crowl T, Estrada-Pinto A, Bithorn J, et al (1998) Do seasonality and disturbance influence reproduction in freshwater atyind shrimp in headwater streams, Puerto Rico? Verh. Int. Ver. Theor. Angew. Limnol. 26: 2076–2081.

[29] Fernández DS, Tremblay RL, Ackerman JD, Rodríguez E, López LN (2003) Reproductive potential, growth rate and light environment in *Lepanthes rupestris* Stimson. Lankersteriana 7: 73–76.

[30] Agosto Pedroza MM, Tremblay RL (2003) El área fotosintética como indicador de la producción de flores en *Lepanthes sanguinea* . Lankesternia 7: 65–66.

[31] Caswell H (2001) Matrix Population Models: Construction, analysis, and interpretation. Sunderland: Sinauer Associates Inc Publishers. pp.722.

[32] Akçakaya HR (2000) Population viability analyses with demographically and spatially structured models. Ecol. Bull 48: 23–38.

[33] Ackerman JD (1995) An Orchid Flora of Puerto Rico and the Virgin Islands. Memoirs of the New York Botanical Gardens. New York: The New York Botanical Garden. pp. 203.

[34] Tremblay RL, Hutchings MJ (2003) Population dynamics in orchid conservation: a review of analytical methods based on the rare species *Lepanthes eltoroensis*. In: Dixon K, Kell S, Barrett R, Cribb P, editors.Orchid Conservation, Borneo: Natural History Museum Publications. pp. 183–204.

[35] Tremblay RL (2000) Plant longevity in four species of *Lepanthes* (Pleurothallidinae; Orchidaceae). Lindleyana 15: 257–266.

[36] Rosa-Fuentes EA, Tremblay RL (2007) Re-evaluation of lifespan in a Neotropical orchid: an eleven years survey. Lankersteriana 7: 204–208.

[37] Spiegelhalter DA, Thomas AN, Best N, Lunn D (2005) WinBUGS User Manual, version 2.10.

[38] Lunn D, Jackson C, Best N, Thomas A, Spiegelhalter D (2012) The BUGS book: A practical introduction to Bayesian Analysis. CRC Press, Boca Raton: Chapman & Hall, Boca Raton. pp. 399.

[39] Kruschke JK (2011) Doing Bayesian Data Analysis: A tutorial with R and BUGS. Oxford: Academic Press, Elsevier. pp. 653.

[40] Bolstad WM (2007) Introduction to Bayesian Statistics, 2^nd^ Hoboken: John Wiley & Sons. pp. 437.

[41] Shefferson RP, Kull T, Tali K (2006) Demographic response to shading and defoliation in two woodland orchids. Folia Geobot 41: 95–116.

[42] Stott I, Franco M, Carslake D, Townley S, Hodgson DJ (2010) Boom or bust? A comparative analysis of transient population dynamics in plants. J. Ecol 98: 302–311.

[43] Stott I, Townley S, Carslake D, Hodgson DJ (2010) On reducibility and ergodicity of population projection matrix models. Methods Ecol. Evol 1: 242–252.

[44] Stott I, Townley S, Hodgson DJ (2011) A framework for studying transient dynamics of population projection matrix models. Ecol. Lett 14: 959–970. 21790932 10.1111/j.1461-0248.2011.01659.x

[45] Cintrón-Berdecia ST, Tremblay RL (2006) Spatial variation in phenotypic selection on floral characteristics in an epiphytic orchid. Folia Geobot 41: 33–46.

[46] Kull T (2002) Population dynamics of North Temperate Orchids. In: Kull T, Arditti, J, editors. Orchid Biology: Review and Perspectives, *VIII*.Dordretch: Kluwer Academic Publishers. pp. 139–165.

[47] Zotz G, Tyree MT (1996) Water stress in the epiphytic orchid, *Dimerandra emarginata* . Oecologia 107: 151–159. 28307300 10.1007/BF00327898

[48] Zotz G (1998) Demography of the epiphytic orchid, *Dimerandra emarginata* . J. Trop. Ecol 14: 725–741.

[49] Zotz G (1999) What are backshoots good for? Seasonal changes in mineral, carbohydrate, and water content of different organs of the epiphytic orchid, *Dimerandra emarginata* . Ann. Bot 84: 791–798.

[50] Zotz G (2000) Size dependence in the reproductive allocation of *Dimerandra emarginata*, an epiphytic orchid. Ecotropica 6: 95–98.

[51] Hernández-Apolinar M (1992) Dinámica poblacional de *Laelia speciosa* (HBK.) Schltr. (Orchidaceae). Tesis de licenciatura, Universidad Nacional Autonoma de Mexico.

[52] Tremblay RL (1997) *Lepanthes caritensis*, an endangered orchid: no sex, no future? Selbyana 18: 160–166.

[53] Tremblay RL, Ackerman JD, Zimmerman JK, Calvo RN (2005) A review of variation in sexual reproduction in orchids and its evolutionary consequences: a spasmodic journey to diversification. Biol. J. Linn. Soc 84: 1–54.

[54] Campbell C, Cousens R (2004) Analysis of monitoring data for *Caladenia versicolor* G. A. Carr. Research Subject 950–612. Masters Thesis, University of Melbourne, Victoria 3010, Australia.

[55] Kéry M, Gregg KB (2004) Demographic analysis of dormancy and survival in terrestrial orchid *Cypripedium reginae* . J. Ecol 92: 686–695.

[56] Silvertown J, Franco M (1993) Plant Demography and Habitat: A comparative Approach. Plant Species Biol 8: 67–73.

[57] Waite S, Hutchings M. (1991) The effects of different management regimes on the population dynamics of *Ophrys sphegodes*: analysis and description using matrix models. In: Wells TCE, Willems JH, editors. Population Ecology of Terrestrial Orchids. The Hague: SPB Academic Publishing bv. pp 161–175.

[58] Ebert TA (1999) Plant and Animal Populations: Methods in Demography. San Diego: Academic Press. pp. 312.

